# Cardiac myosin-binding protein C is a novel marker of myocardial injury and fibrosis in aortic stenosis

**DOI:** 10.1136/heartjnl-2017-312257

**Published:** 2017-12-01

**Authors:** Atul Anand, Calvin Chin, Anoop S V Shah, Jacek Kwiecinski, Alex Vesey, Joanna Cowell, Ekkehard Weber, Thomas Kaier, David E Newby, Marc Dweck, Michael S Marber, Nicholas L Mills

**Affiliations:** 1 BHF Centre for Cardiovascular Science, University of Edinburgh, Edinburgh, UK; 2 First Department of Cardiology, Poznan University of Medical Sciences, Poznan, Poland; 3 Department of Geriatric Medicine, Royal Victoria Building, Edinburgh, UK; 4 Institute of Physiological Chemistry, Martin Luther University Halle-Wittenberg, Halle, Germany; 5 King’s College London BHF Centre, The Rayne Institute, St Thomas’ Hospital, London, UK

**Keywords:** aortic stenosis, cardiac magnetic resonance (CMR) imaging

## Abstract

**Objective:**

Cardiac myosin-binding protein C (cMyC) is an abundant sarcomeric protein and novel highly specific marker of myocardial injury. Myocyte death characterises the transition from hypertrophy to replacement myocardial fibrosis in advanced aortic stenosis. We hypothesised that serum cMyC concentrations would be associated with cardiac structure and outcomes in patients with aortic stenosis.

**Methods:**

cMyC was measured in two cohorts in which serum had previously been prospectively collected: a mechanism cohort of patients with aortic stenosis (n=161) and healthy controls (n=46) who underwent cardiac MRI, and an outcome cohort with aortic stenosis (n=104) followed for a median of 11.3 years.

**Results:**

In the mechanism cohort, cMyC concentration correlated with left ventricular mass (adjusted Î²=11.0 g/m^2^ per log unit increase in cMyC, P<0.001), fibrosis volume (adjusted Î²=8.0 g, P<0.001) and extracellular volume (adjusted Î²=1.3%, P=0.01) in patients with aortic stenosis but not in controls. In those with late gadolinium enhancement (LGE) indicative of myocardial fibrosis, cMyC concentrations were higher (32 (21–56) ng/L vs 17 (12–24) ng/L without LGE, P<0.001). cMyC was unrelated to coronary calcium scores. Unadjusted Cox proportional hazards analysis in the outcome cohort showed greater all-cause mortality (HR 1.49 per unit increase in log cMyC, 95% CI 1.11 to 2.01, P=0.009).

**Conclusions:**

Serum cMyC concentration is associated with myocardial hypertrophy, fibrosis and an increased risk of mortality in aortic stenosis. The quantification of serum sarcomeric protein concentrations provides objective measures of disease severity and their clinical utility to monitor the progression of aortic stenosis merits further study.

**Clinical trial registration:**

NCT1755936; Post-results.

## Introduction

Aortic stenosis is the most common valvular disease in the Western world and the incidence is rising in keeping with an ageing population.^1^ The response of the myocardium to aortic stenosis is variable, with heterogeneity in the development of ventricular hypertrophy and in how this process ultimately decompensates.^2–4^ This results in a poor correlation between the severity of stenosis and the development of symptoms. Decompensation of the hypertrophic response in aortic stenosis is driven by two processes: progressive myocyte cell death and myocardial fibrosis.^5^ Biomarkers of myocardial injury are therefore an attractive addition to current imaging markers of disease progression, perhaps providing critical evidence of early decompensation that may identify patients who would benefit from early valve replacement. We have previously demonstrated that cardiac troponin I concentration is associated with advanced hypertrophy, replacement fibrosis and poor long-term outcomes in patients with aortic stenosis,^6^ suggesting that myocardial injury in advanced aortic stenosis is common, detectable and of prognostic importance.

Cardiac myosin-binding protein C (cMyC) is a cardiac-restricted sarcomeric protein located on the thick filament.^7 8^ Recent advances in assay technology have allowed measurement of cMyC with high precision at extremely low concentrations.^9^ It is more abundant in myocardial tissue and the circulation than cardiac troponins and has an important role in the assembly and function of the cardiac sarcomere.^10–12^ Indeed, mutations in the *MYBPC3* gene-encoding cMyC are the most common known genetic cause of hypertrophic cardiomyopathy.^13^ Furthermore, the proteolytic cleavage of cMyC is highly regulated by a variety of myocardial kinases^14^ and the resultant peptide is cardiotoxic.^15^ Hence, there is growing interest in the protein as both a biomarker and a determinant of myocardial injury.^16^ Given myocyte death characterises the transition from hypertrophy to replacement myocardial fibrosis in advanced aortic stenosis, we hypothesised that serum cMyC concentrations would be associated with cardiac structure and outcomes in patients with aortic stenosis.

## Methods

We evaluated cMyC in two cohorts: a *mechanism* cohort of patients with aortic stenosis and healthy controls with cardiac MRI (CMR), and an *outcome* cohort of patients with aortic stenosis with more than 10 years of clinical follow-up. These groups were derived from existing studies in patients with stable aortic stenosis recruited from cardiology clinics across the South East of Scotland, where serum had been prospectively collected and frozen at the time of study inclusion. Additionally, a subgroup of patients from the mechanism cohort underwent myocardial biopsy at the time of subsequent aortic valve replacement (AVR), providing exploratory histological correlation with cMyC concentrations. The studies were conducted in accordance with the Declaration of Helsinki. Written informed consent was obtained from all participants.

### Mechanism cohort

The mechanism cohort consisted of 161 patients with mild to severe aortic stenosis and 46 healthy volunteers without evidence of significant valvular heart disease enrolled in an observational study assessing the role of myocardial fibrosis in aortic stenosis (NCT:01755936 post-results). Exclusion criteria comprised significant (moderate or severe) non-aortic valvular disease or any cardiomyopathy (acquired or inherited). The imaging protocol undertaken in all participants has been described in detail previously.^6 17–19^ Briefly, CMR was performed using a 3T scanner (MAGNETOM Verio, Siemens AG, Healthcare Sector, Erlangen, Germany). Dedicated software was used to assess left ventricular (LV) volume and mass indexed to body surface area and to calculate ejection fraction. Diffuse myocardial fibrosis was determined by fibrosis volume and the extracellular volume (ECV) fraction in keeping with current evidence of reproducibility from T1 mapping.^19 20^ Focal myocardial replacement fibrosis was determined by the late gadolinium enhancement (LGE) technique, with its presence determined visually and independently by two experienced assessors. Comprehensive echocardiography was performed on all subjects to classify markers of aortic stenosis severity according to European Association of Echocardiography/American Society of Echocardiography guidelines.^21^

### Outcome cohort

This cohort was derived from the Scottish Aortic Stenosis and Lipid Lowering Trial, Impact of Regression (SALTIRE) study. The study design, recruitment and findings have been reported previously.^22 23^ Briefly, between March 2001 and April 2002, a total of 155 patients with asymptomatic moderate to severe aortic stenosis were randomised to receive either atorvastatin or placebo. Stored sample remained for cMyC analysis in 104 patients. In addition to comprehensive echocardiography, CT calcium scoring of the coronary arteries was performed (Twin II Flash, Philips Medical Systems).

Outcomes data were obtained by two independent investigators who were blinded to cMyC results. The General Register of Scotland was searched for all deaths. The cause of death was adjudicated for a cardiac cause using additional information from electronic health records if necessary. Disagreements were resolved by consensus. Electronic health records were also reviewed in all cases for evidence of surgical AVR.

### Blood sampling and analysis

Serum cMyC concentrations were measured in duplicate on the single molecule counting Erenna platform (Singulex/Merck Millipore, CA, USA). The assay has a lower limit of detection (LoD) of 0.4 ng/L, a lower limit of quantification (LLoQ) of 1.2 ng/L (at 20% coefficient of variation, CV) and reasonable recovery (107.1%±3.7%; mean±SD), dilutional linearity (101.0%±7.7%), and intraseries (CV 11%±3%) and interseries (CV 13%±3%) precision. Cardiac troponin was determined using a high-sensitivity assay (Abbott ARCHITECT STAT, Abbott Diagnostics, IL, USA) as previously described. This assay has an LoD of 1.2 ng/L and based on our previous work an LoQ of 1.5 ng/L (at 20% CV).^6^

### Myocardial biopsy and histological analysis

Tru-cut myocardial biopsies were obtained from 10 patients in the mechanism cohort who underwent AVR and also had cMyC measured. For autophagy and oncosis assessment, formalin-fixed, paraffin-embedded 4 µm thick tissue sections were cut and dehydrated. Further details of the histological analysis are provided in the online supplementary material.

10.1136/heartjnl-2017-312257.supp1 Supplementary file 1

### Statistical analysis

Statistical analysis was performed using the statistical software R V.3.3.2 (http://www.r-project.org). Continuous variables are presented as mean (SD) or median (IQR) for non-parametric data. We used analysis of variance to compare continuous parametric data and the Kruskal-Wallis test for non-parametric data across tertiles of cMyC. Categorical variables are presented as absolute numbers (percentage) and were compared using χ^2^ test. Due to the positive skewing in the sample, cMyC concentrations were log transformed prior to inclusion in modelling. Multivariate linear regression modelling was used to assess the change in markers of aortic stenosis severity with serum cMyC concentration. Receiver operating characteristic (ROC) curve analysis and multivariate logistic regression modelling were used to assess the relationship between cMyC concentrations and LGE. Survival analysis was performed with a Kaplan-Meier analysis using time to any cause of death, with significance calculated by log-rank test. This relationship was also assessed using Cox proportional hazard modelling. AVR was included as a time-varying covariate as survival could be expected to improve in those who received surgery during the follow-up period. A value of P<0.05 was considered statistically significant.

## Results

The mechanism cohort consisted of 161 individuals with aortic stenosis (mean age 69 years, AV_max_ 3.8±0.9 m/s, 70% male) and 46 healthy control participants (mean age 58 years, AV_max_ 1.4±0.2 m/s, 63% male). The outcome cohort consisted of 104 patients (mean age 68 years, AV_max_ 3.4±0.7 m/s, 68% male) with a median follow-up period of just more than 11 years (4067 (3882–4161) days).

Baseline characteristics by tertile of cMyC are presented in tables 1 and 2 for the mechanism and outcome cohorts. cMyC was measurable above the LLoQ (1.2 ng/L) in all but one subject, giving an overall detection rate of 99.7%, including all healthy controls (online supplementary figure S1). Similar to observations with cardiac troponin, cMyC concentrations were positively skewed across all cohorts (online supplementary figure S2) and weakly correlated with renal function (online supplementary figure S3). The median cMyC concentration was similar among patients with aortic stenosis in the mechanism (20.5 (13.7–33.2) ng/L) and outcome (18.2 (12.2–30.1) ng/L) cohorts (P=0.21), with both being higher than in controls (9.5 (7.6–15.1) ng/L, P<0.001 for both). Samples were tested in duplicate, with a CV between repeated measurements of 5.9%±5.1% in the mechanism cohort, 6.1%±4.9% across the outcome cohort and 8.5%±4.5% in controls.

**Table 1:** Baseline characteristics of the mechanism cohort by tertile of cMyC and controls

	All (n=161)	Tertile 1 3–16 ng/L (n=54)	Tertile 2 17–28 ng/L (n=55)	Tertile 3 29–171 ng/L (n=52)	P value	Controls (n=46)
Age (years)	68.5 (11.4)	64.3 (13.0)	69.4 (9.3)	71.8 (10.5)	**0.002**	57.9 (20.5)
Sex (male), n (%)	112 (69.6)	29 (53.7)	42 (76.4)	41 (78.8)	**0.008**	29 (63.0)
BMI (kg/m^2^)	28.9 (4.8)	29.1 (5.2)	28.7 (4.0)	29.0 (5.1)	0.88	26.8 (3.8)
BSA (m^2^)	1.9 (0.2)	1.9 (0.2)	1.9 (0.2)	1.9 (0.2)	0.71	1.9 (0.2)
Comorbidity						
Diabetes mellitus, n (%)	24 (14.9)	9 (16.7)	6 (10.9)	9 (17.3)	0.59	0 (0.0)
Hypertension, n (%)	109 (67.7)	30 (55.6)	42 (76.4)	37 (71.2)	0.06	12 (26.1)
Hyperlipidaemia, n (%)	72 (44.7)	20 (37.0)	22 (40.0)	30 (57.7)	0.07	8 (17.4)
IHD, n (%)	61 (37.9)	12 (22.2)	23 (41.8)	26 (50.0)	**0.01**	2 (4.3)
Previous PCI, n (%)	25 (15.5)	6 (11.1)	8 (14.5)	11 (21.2)	0.35	2 (4.3)
Previous CABG, n (%)	7 (4.4)	2 (3.7)	1 (1.9)	4 (7.7)	0.33	0 (0.0)
Medications						
Antiplatelet, n (%)	79 (49.1)	17 (31.5)	29 (52.7)	33 (63.5)	**0.004**	7 (15.2)
ACE-I/ARB, n (%)	67 (41.6)	16 (29.6)	28 (50.9)	23 (44.2)	0.07	9 (19.6)
Beta blocker, n (%)	55 (34.2)	16 (29.6)	17 (30.9)	22 (42.3)	0.32	5 (10.9)
Diuretic, n (%)	52 (32.3)	16 (29.6)	21 (38.2)	15 (28.8)	0.51	4 (8.7)
Blood tests						
Creatinine (μmol/L)	79.1 (17.1)	73.5 (11.1)	78.6 (15.5)	85.2 (21.5)	**0.002**	72.5 (15.3)
eGFR (mL/min/1.73 m^2^)	86.3 (18.5)	89.0 (15.9)	88.0 (18.8)	81.7 (20.1)	0.09	97.7 (19.8)
Troponin I (ng/L)	6.6 (3.8–12.3)	3.3 (2.3–4.5)	6.7 (4.8–9.5)	15.5 (10–30.2)	**<0.001**	2.7 (1.1–5.4)
Echo parameters						
AV_max_ (m/s)	3.8 (0.9)	3.4 (0.8)	3.9 (0.8)	4.3 (0.9)	**<0.001**	1.4 (0.2)
AV area (cm^2^)	1.0 (0.4)	1.1 (0.4)	0.9 (0.3)	0.9 (0.4)	**0.006**	2.4 (0.6)
AV MPG (mm Hg)	34.4 (18.6)	25.9 (13.5)	34.5 (16.6)	43.2 (21.3)	**<0.001**	4.2 (1.4)
Indexed LV mass (g/m^2^)	122.7 (32.0)	102.3 (22.8)	127.6 (27.3)	139.4 (33.7)	**<0.001**	93.2 (24.4)
E/e′	14.6 (7.6)	12.4 (4.8)	13.1 (5.0)	18.3 (10.4)	**<0.001**	8.6 (2.5)
MRI parameters						
Indexed LV mass (g/m^2^)	88.8 (21.5)	74.2 (15.2)	90.1 (16.6)	102.5 (22.1)	**<0.001**	65.8 (13.7)
Ejection fraction (%)	66.8 (7.3)	67.5 (5.6)	65.8 (8.3)	67.2 (7.8)	0.44	64.4 (4.4)
Indexed SV (mL/m^2^)	47.6 (10.2)	44.3 (8.4)	47.1 (9.6)	51.4 (11.2)	**0.001**	46.1 (8.2)
Indexed ESV (mL/m^2^)	24.2 (9.8)	21.6 (6.6)	25.5 (11.5)	25.6 (10.2)	0.055	26.0 (7.4)
Indexed EDV (mL/m^2^)	71.8 (16.9)	65.9 (13.3)	72.6 (17.8)	77.0 (17.8)	**0.003**	72.1 (14.6)
ECV (%)	27.8 (2.6)	27.1 (2.2)	27.4 (2.1)	28.9 (3.2)	**0.001**	26.6 (1.7)
Fibrosis volume (g)	44.6 (15.3)	36.0 (8.9)	44.2 (11.1)	54.5 (18.5)	**<0.001**	31.0 (7.6)
Outcomes						
AVR, n (%)	41 (25.5)	8 (14.8)	20 (36.4)	13 (25.0)	**0.04**	0 (0.0)
Cardiac death, n (%)	1 (0.6)	0 (0.0)	0 (0.0)	1 (1.9)	0.35	0 (0.0)
All-cause death, n (%)	6 (3.7)	1 (1.9)	1 (1.8)	4 (7.7)	0.19	0 (0.0)

Values are number (%), mean (SD) or median (IQR). Results in bold are statistically significant.

ACE-I, ACE inhibitor; ARB, angiotensin receptor blocker; AV, aortic valve; AVR, aortic valve replacement.; BMI, body mass index; BSA, body surface area; CABG, coronary artery bypass grafting; cMyC, cardiac myosin-binding protein C; ECV, extracellular volume fraction; EDV, end-diastolic volume; eGFR, estimated glomerular filtration rate; ESV, end-systolic volume; IHD, ischaemic heart disease; PCI, percutaneous coronary intervention; LV, left ventricle; MPG, mean pressure gradient; SV, stroke volume.

**Table 2:** Baseline characteristics of the outcome cohort by tertile of cMyC

	All patients (n=104)	Tertile 1 5–14 ng/L (n=35)	Tertile 2 15–26 ng/L (n=35)	Tertile 3 27–132 ng/L (n=34)	P value
Age (years)	68.2 (9.8)	64.4 (10.5)	70.1 (9.0)	70.0 (9.1)	**0.02**
Sex (male), n (%)	71 (68.3)	21 (60.0)	19 (54.3)	31 (91.2)	**0.002**
BMI (kg/m^2^)	27.9 (4.5)	27.6 (4.1)	28.1 (4.9)	27.8 (4.7)	0.91
BSA (m^2^)	1.9 (0.2)	1.9 (0.2)	1.9 (0.2)	2.0 (0.2)	0.19
Comorbidity					
Diabetes mellitus, n (%)	2 (1.9)	1 (2.9)	0 (0.0)	1 (2.9)	0.60
Hypertension, n (%)	57 (54.8)	16 (45.7)	24 (68.6)	17 (50.0)	0.13
Hyperlipidaemia, n (%)	7 (6.7)	0 (0.0)	5 (14.3)	2 (5.9)	0.06
IHD, n (%)	17 (16.3)	2 (5.7)	10 (28.6)	5 (14.7)	**0.03**
Medications					
Aspirin, n (%)	42 (40.4)	13 (37.1)	16 (45.7)	13 (38.2)	0.74
Anticoagulant, n (%)	10 (9.6)	1 (2.9)	4 (11.4)	5 (14.7)	0.23
ACE-I/ARB, n (%)	20 (19.2)	5 (14.3)	7 (20)	8 (23.5)	0.63
Beta blocker, n (%)	23 (22.1)	8 (22.9)	11 (31.4)	4 (11.8)	0.14
Blood tests					
Creatinine (μmol/L)	91.3 (21.3)	85.3 (18.1)	89.3 (17.9)	100.1 (25.6)	0.01
eGFR (mL/min/1.73 m^2^)	73.5 (17.4)	77.2 (13.9)	71.7 (16.4)	71.6 (21.3)	0.31
Troponin I (ng/L)	7.2 (5.4–12.6)	4.9 (3.9–6.0)	8.3 (6.7–10.6)	14.3 (12.1–17.9)	**<0.001**
Echo parameters					
AV_max_ (m/s)	3.4 (0.7)	3.4 (0.7)	3.4 (0.8)	3.5 (0.6)	0.90
AV area (cm^2^)	1.0 (0.4)	1.0 (0.4)	1.0 (0.4)	1.0 (0.4)	0.89
AV MPG (mm Hg)	48.8 (19.2)	47.6 (18.7)	49.7 (21.8)	49.2 (17.1)	0.89
Indexed LV mass (g/m^2^)	184.4 (52.2)	170.0 (56.8)	175.7 (38.8)	208.7 (52.0)	**0.004**
Ejection fraction (%)	66 (31)	69 (22)	66 (25)	61 (44)	0.64
CT coronary calcium score	382.6 (611.1)	370.3 (608.3)	491.0 (783.2)	283.6 (360.2)	0.37
Outcomes					
AVR, n (%)	48 (46.2)	19 (54.3)	15 (42.9)	14 (41.2)	0.49
Cardiac death, n (%)	16 (15.4)	4 (11.4)	7 (20.0)	5 (14.7)	0.61
All-cause death, n (%)	36 (34.6)	7 (20.0)	14 (40.0)	15 (44.1)	0.08

Values are number (%), mean (SD) or median (IQR). Results in bold are statistically significant.

ACE-I, ACE inhibitor; ARB, angiotensin receptor blocker; AV, aortic valve; AVR, aortic valve replacement; BMI, body mass index; BSA, body surface area; cMyC, cardiac myosin-binding protein C; eGFR, estimated glomerular filtration rate; IHD, ischaemic heart disease; LV, left ventricle; MPG, mean pressure gradient.

Cardiac troponin concentrations were above the LLoQ in 92.3% of subjects, but in only 65.2% of healthy controls. A strong correlation was observed between cMyC and cardiac troponin across all samples tested (r=0.74, 95% CI 0.69 to 0.79, P<0.0001, online supplementary figure S1).

### cMyC and mechanistic imaging markers

**Figure 1:**
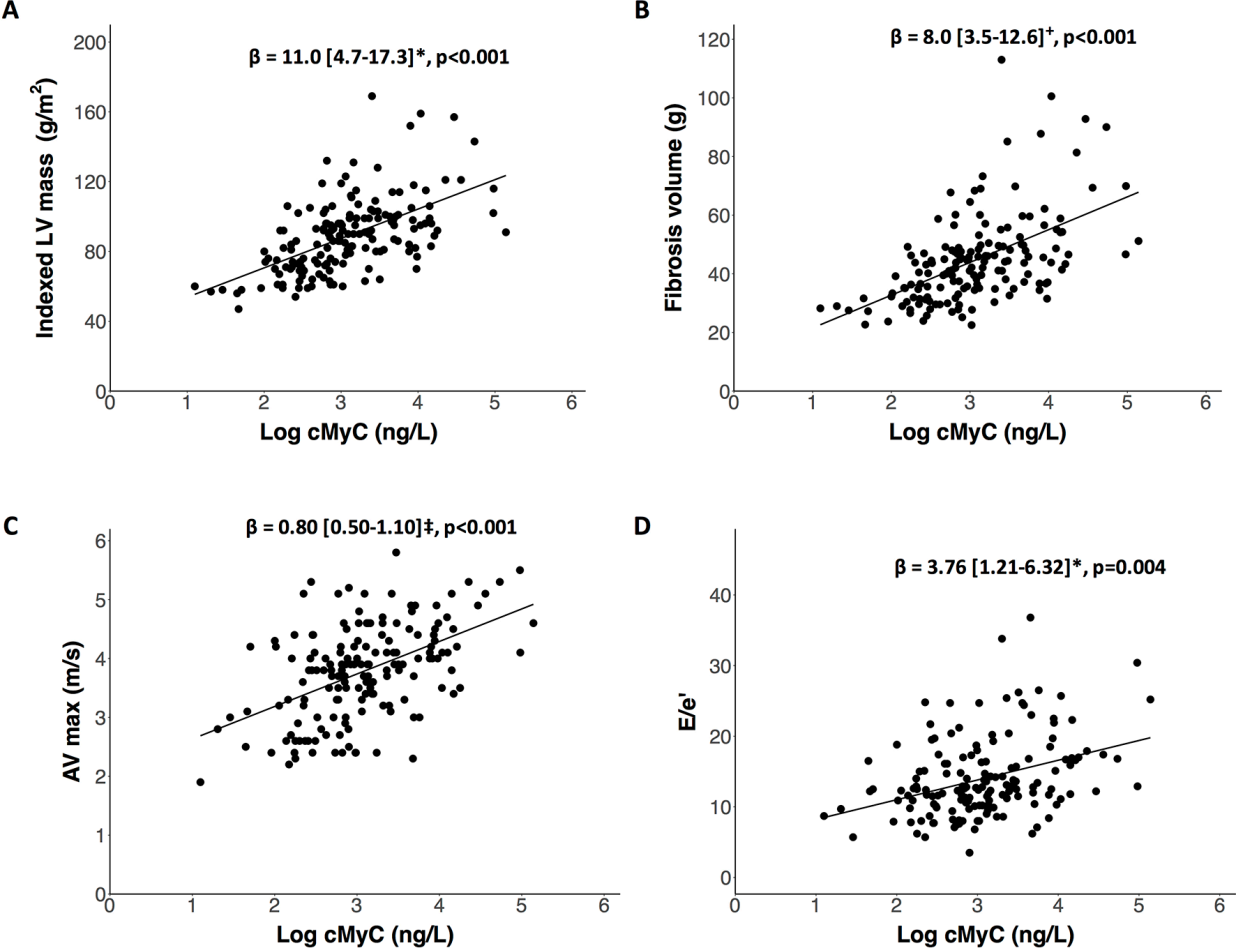
Relationships between cMyC and markers of disease severity in the mechanism cohort. β values (95% CI) represent change in the progression variable for each log unit change in cMyC concentration after adjustment in multivariate modelling. Markers of disease severity are: (A) indexed LV mass (g/m^2^) determined by MRI; (B) fibrosis volume (g) by MRI; (C) AV_max_ (m/s) determined by echocardiography; (D) diastolic function from echocardiographic measures of E/e′ ratio. *Adjusted for age, sex, glomerular filtration rate, AV_max_, cardiac troponin, history of ischaemic heart disease, diabetes and hypertension. +As above plus body surface area. ‡As above excluding AV_max_. AV, aortic valve; cMyC, cardiac myosin-binding protein C; LV, left ventricle.

In adjusted multiple regression analyses, cMyC concentration was associated with indexed LV mass in those with aortic stenosis who underwent CMR imaging (β=11.0 g/m^2^ per log unit increase in cMyC after adjustment for age, sex, renal function, AV_max_, cardiac troponin and comorbidity; 95% CI 4.7 to 17.3, P<0.001, figure 1A). This relationship was numerically positive, but did not approach statistical significance among healthy controls (β=2.7 g/m^2^, 95% CI −4.8 to 10.1, P=0.47, online supplementary figure S4). Similarly, fibrosis volume was related to cMyC in those with aortic stenosis (adjusted β=8.0 g per log unit increase in cMyC; 95% CI 3.5 to 12.6, P<0.001, figure 1B) but not in controls (β=1.8 g, 95% CI −1.6 to 5.2, P=0.28, online supplementary figure S4). ECV as a marker of diffuse myocardial fibrosis was associated with cMyC in patients with aortic stenosis (adjusted β=1.3%, 95% CI 0.3% to 2.3%, P=0.01), but not in healthy controls (adjusted β=0.5%, 95% CI −0.5% to 1.5%, P=0.32). Detailed modelling is shown in online supplementary tables S1–S3. cMyC concentrations were related to severity of aortic stenosis across the range of AV_max_ measures in the mechanism cohort (adjusted β=0.80 m/s, 95% CI 0.50 to 1.10, P<0.001, figure 1C), although this was not observed in the narrower range included in the outcome cohort (online supplementary figure S4). An association was further noted between cMyC and diastolic function, measured by echocardiography E/e′ in the mechanism cohort (adjusted β=3.76, 95% CI 1.21 to 6.32, P=0.004, figure 1D). There was no relationship between cMyC and objective measures of coronary disease by CT calcium scoring (adjusted β=0.03, 95% CI −0.17 to 0.23, P=0.76, online supplementary figure S4).

**Figure 2:**
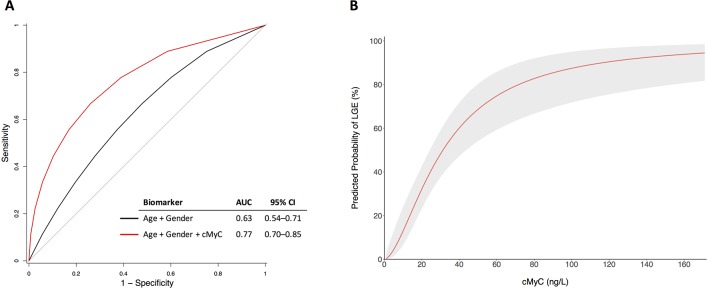
cMyC and late gadolinium enhancement. (A) Receiver operating characteristic (ROC) curve analysis for outcome of LGE. Addition of cMyC improves the prediction of age and gender for LGE, P=0.002 (DeLong’s method with bootstrapping). (B) Logistic regression modelling for the predicted probability of LGE with serum cMyC concentration (adjusted for age and sex). Shaded area represents 95% CI. AUC, area under the curve; cMyC, cardiac myosin-binding protein C; LGE, late gadolinium enhancement.

LGE was present in 57 (35.4%) of patients with aortic stenosis in the mechanism cohort. cMyC concentrations were almost double in those with evidence of LGE compared with those without (32.3 (21.3–56.3) ng/L vs 17.2 (11.5–24.2) ng/L, P<0.001). By ROC analysis, cMyC improved discrimination for LGE beyond age and gender (area under the curve (AUC) 0.77, 95% CI 0.70 to 0.85, figure 2A). A sensitivity analysis restricted to those with mid-wall fibrosis only (77% of those with any LGE) demonstrated similar discrimination (AUC 0.77, 95% CI 0.70 to 0.84). Using logistic regression modelling adjusted for age and sex, the predicted probability of LGE was seen to progressively increase with cMyC concentration (figure 2B). Univariate correlations between cMyC and baseline variables used for covariate adjustment in the mechanism cohort are presented in online supplementary table S4.

### cMyC and myocyte death

**Figure 3:**
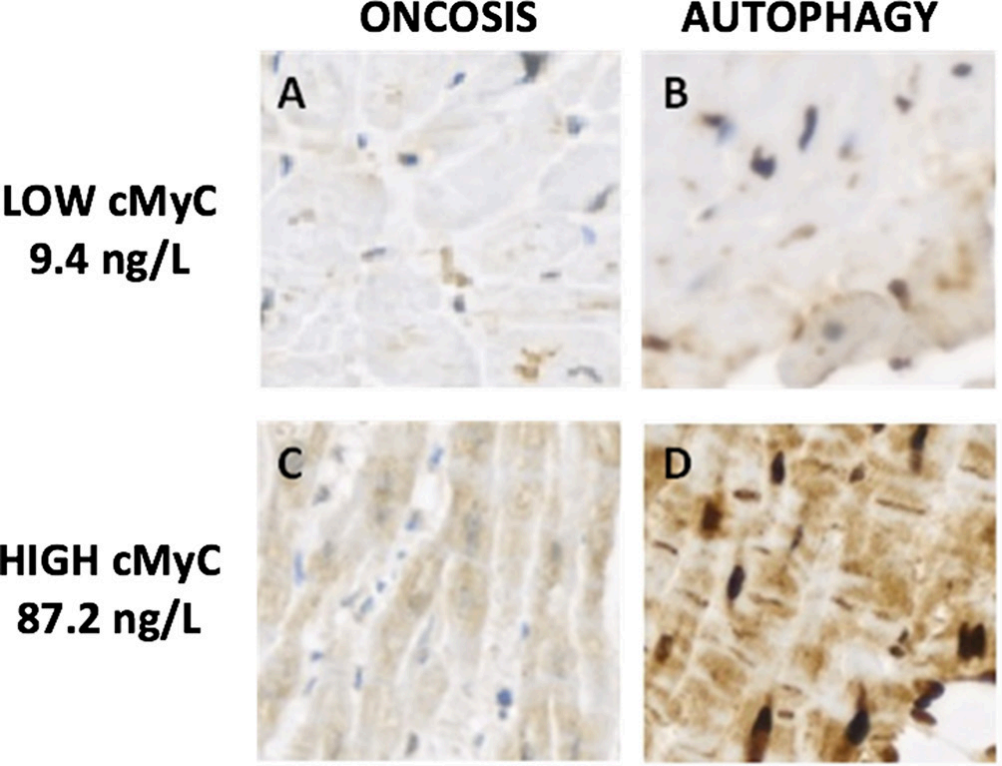
Patterns of myocyte death in patients with aortic stenosis. Images showing differing patterns of *oncosis* and *autophagy* visualised using a 3,3′-diaminobenzidine-based detection kit in individuals with aortic stenosis. (A) *Oncosis* and (B) *autophagy* in a patient with a low (9.4 ng/L) cMyC concentration. (C) *Oncosis* and (D) *autophagy* in a patient with a high (87.2 ng/L) cMyC concentration. There is a marked difference in staining intensity by cMyC concentration for both oncosis and autophagy. cMyC, cardiac myosin-binding protein C.

Clear differences were observed in staining patterns for oncosis and autophagy between subjects with low and high cMyC concentrations (figure 3). Exploratory analysis suggested a relationship between cMyC and the rate of myocyte death (expressed as the sum of apoptosis, oncosis and autophagy counts) in 10 subjects with myocardial biopsy tissue taken at the time of AVR (r=0.67, 95% CI 0.08 to 0.92, P=0.03, online supplementary figure S5).

### cMyC and long-term outcomes

**Figure 4:**
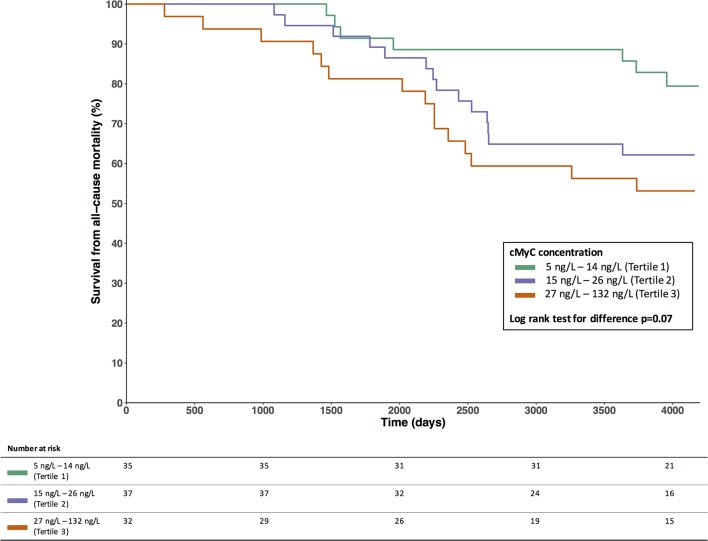
Survival by tertiles of cMyC in the outcome cohort. cMyC, cardiac myosin-binding protein C.

Subjects in the outcome cohort were stratified by tertile of cMyC (table 2). As with CMR in the mechanism cohort, indexed LV mass by echocardiography increased across tertiles of cMyC (170.0±56.8 g in lowest tertile vs 208.7±52.0 g in highest tertile, P=0.004). There were no consistent differences in cardiac risk factors across the tertiles. During the follow-up period, 36 (34.6%) subjects died, of which 16 (15.4%) were adjudicated as cardiac deaths. Forty-eight patients (46.2%) within the cohort underwent surgical AVR, with no cases of transcatheter aortic valve implantation undertaken. There was a trend towards poorer survival over the period of follow-up with increasing tertile of cMyC (figure 4, log-rank test for difference P=0.07).

In Cox proportional hazards analysis, cMyC concentration was associated with an increased risk of all-cause mortality over the follow-up period after inclusion of AVR as a time-varying covariate (HR 1.49 per log unit increase of cMyC, 95% CI 1.11 to 2.01, P=0.009). However, following adjustment for age, sex, AV_max_ or CT coronary calcium scores, statistical significance was lost. Age was significantly associated with cMyC in all models (online supplementary table S5).

## Discussion

In a comprehensive series of clinical assessments, we report the relationship between serum cMyC concentration, a novel marker of myocardial injury, and cardiac structure and outcomes in patients with aortic stenosis. We have made several important observations. First, serum cMyC concentrations can be reliably quantified in the vast majority of patients with aortic stenosis. Second, cMyC strongly associates with markers of both diffuse interstitial and focal replacement fibrosis as well as indexed LV mass, a marker of the hypertrophic response to aortic stenosis. These observations were independent of the severity of valve stenosis and cMyC concentrations were unrelated to coronary artery disease. Third, cMyC concentrations are strongly correlated with cardiac troponin, and in histological analyses are associated with myocyte cell death, suggesting that sarcomeric protein release is a direct consequence of the maladaptive myocardial response to aortic stenosis. We believe these novel observations demonstrate the utility and validity of cMyC as a marker of sustained chronic myocardial injury.

### Assay performance

The release profile of cMyC has been previously studied after acute injury in myocardial infarction, alcohol ablation for hypertrophic cardiomyopathy and coronary artery bypass grafting.^7 16^ These models have shown cMyC to be more abundant, to be released earlier following injury and to decline more rapidly when compared with cardiac troponin. These observations suggest that cMyC has enhanced potential for dynamic monitoring of myocardial injury and disease. However, previous generations of the cMyC assay had limited sensitivity at the low levels that would be expected in the presence of chronic myocardial injury such as in aortic stenosis. For example, cMyC was only detectable in 2 out of 20 patients with hypertrophic cardiomyopathy.^24^ Here we report cMyC measurements from a high-sensitivity assay using magnetic nanoparticle and single molecule counting technology, and demonstrate near universal quantification in patients with aortic stenosis and healthy controls.

### cMyC as a biomarker of disease

Our observations suggest cMyC is a sensitive marker of the hypertrophic and subsequent fibrotic myocardial response to aortic stenosis. ECV fraction on CMR T1 mapping is a marker of diffuse interstitial fibrosis, which may precede the focal mid-wall replacement fibrosis detected by LGE.^19 20^ Histological studies have suggested that myocyte death drives this transition.^5^ Our exploratory analysis in myocardial biopsy tissue relates combined measures of autophagy, oncosis and apoptosis to serum cMyC concentrations, providing histological correlation of biomarker release as a result of myocyte death. This combination of autophagy and oncosis in failing myocardial tissue has been independently associated with mortality in larger studies.^25^

Our observations are consistent with our previous findings using a high-sensitivity cardiac troponin I assay. Using two independent sarcomeric proteins, we have now demonstrated that serum markers of myocardial injury mirror important pathological changes in the myocardium in response to aortic stenosis.^6 26 27^ Similar to cardiac troponin, although cMyC was related to AV_max_ by echocardiography in the mechanism cohort, its association with markers of hypertrophy, fibrosis and diastolic function was independent of the severity of aortic stenosis. This suggests that these novel markers of the myocardial response to stenosis will add to conventional clinical assessment in aortic stenosis. While CMR imaging provides important detail on the extent of the progression to fibrosis and heart failure, it is a resource-intensive investigation.^2 28^ As such, there is great potential for simple blood measures of myocardial injury to improve decision-making in aortic stenosis, to better target detailed imaging or even help determine the timing of surgery. Current guidelines advocate AVR in the presence of LV impairment or symptoms, but these are challenging to define objectively and may signify that irreversible myocardial fibrosis has already developed. A precise and early marker of myocardial injury such as cMyC may have great clinical utility in patients with aortic stenosis.

We demonstrate an increased mortality risk with rising cMyC concentrations, but this association was not independent of other variables. Our outcome cohort benefits from extensive follow-up over 11 years after sampling, but the cohort size was restricted by limited remaining stored samples. This analysis is likely to be underpowered and further work is required in larger studies to clarify whether cMyC will provide additional prognostic information in the assessment of patients with aortic stenosis.

### Limitations

We acknowledge some limitations with the current study in addition to sample size. There is only limited overlap for comparison of imaging endpoints between our two cohorts, although the magnitude of associations between cMyC and LV mass are similar. The clinical utility of biomarkers such as cMyC is more likely to be of interest in severe cases of aortic stenosis approaching the need for valve replacement, where myocardial injury becomes more pronounced. Our mechanism and outcome cohorts contained only 84 and 52 patients with severe aortic stenosis, respectively. While this represents half of our study population, further evaluation in a larger population with severe disease would be informative. The histological analysis presented is exploratory and the Tru-cut technique used may provide limited volumes of myocardium compared with novel scalpel methods.^29^

Our findings add to our previous observations with cardiac troponin.^6 26 27^ Compared with those assays, the latest generation cMyC assay is still in the early stages of development, with no definitive healthy population studies to derive a normal reference range. However, our study suggests that the biomarker will be measurable in virtually all individuals and is likely to meet the criteria for a high-sensitivity assay.^30^ Larger cohort studies are necessary to assess the relationship and potential differences in cMyC and cardiac troponin release, and to determine whether the association of cMyC and long-term outcomes is independent of other patient factors and cardiac biomarkers. Further validation with prospective serial sampling of cMyC in patients with aortic stenosis prior to clinical decompensation is now required.

## Conclusions

Serum cMyC concentration is associated with myocardial hypertrophy, fibrosis and an increased risk of mortality in aortic stenosis. The quantification of serum sarcomeric protein concentrations provides objective measures of disease severity and their clinical utility to monitor the progression of aortic stenosis merits further study.

**Key messages**

**What is already known on this subject?**

The progression of aortic stenosis is unpredictable and irreversible myocardial fibrosis may develop before symptoms are apparent. Cardiac troponin I as a biomarker of myocardial injury is associated with advanced hypertrophy, replacement fibrosis and poor long-term outcomes in patients with aortic stenosis.

**What might this study add?**

Cardiac myosin-binding protein C (cMyC) is a cardiac sarcomeric protein and novel highly sensitive biomarker of acute myocardial injury. In two cohorts of patients with aortic stenosis, increasing concentrations of cMyC were associated with greater myocardial hypertrophy, fibrosis and death, even after adjustment for the severity of aortic stenosis and cardiac troponin concentrations. This is the first study to measure cMyC using a high-precision assay in patients with aortic stenosis.

**How might this impact on clinical practice?**

Objective blood biomarkers are not currently included in clinical guidelines for the management of aortic stenosis. This study adds to evidence that quantification of serum sarcomeric proteins has major potential to objectively track disease progression in this condition. Further validation with prospective serial sampling of cMyC in patients with aortic stenosis prior to clinical decompensation is now required.
