# Supplementary material for: Cardiac myosin-binding protein C is a novel marker of myocardial injury and fibrosis in aortic stenosis
Source: Heart. 2017 Dec 1;104(13):1101–8. doi: 10.1136/heartjnl-2017-312257 (PMC6031261; doi:10.1136/heartjnl-2017-312257)

SUPPLEMENTAL MATERIAL

Cardiac myosin-binding protein C is a novel marker of myocardial injury and fibrosis in aortic stenosis

Supplemental Figure Legends

Figure S1: Assay performance of the cMyC assay against high-sensitivity cardiac troponin I. (A) Proportion of patients with aortic stenosis (mechanism and outcome cohorts) and (B) controls (mechanism cohort) in whom cardiac troponin and cMyC concentrations were above the lower limit of quantification (20% CV) of 1.5 ng/L and 1.2 ng/L respectively. (C) Correlation between cardiac troponin I and cMyC concentrations across all cohorts (aortic stenosis and control patients).

Figure S2: Frequency histograms of cMyC concentrations measured in each cohort and in all samples tested, demonstrating similarly positively skewed distributions in each case. cMyC was detectable above the lower limit of quantification in 99.7% of samples tested.

Figure S3: Relationship between renal function and sarcomeric proteins– correlation between cMyC (left) and cTnI (right) and estimated glomerular filtration rate across all patients with aortic stenosis and controls. Pearson correlation coefficient (r) presented with 95% confidence interval.

Figure S4: Linear regression correlations cMyC concentration with (A) Indexed LV mass (grams/m^2) in control patients determined by MRI (mechanism cohort); (B) Fibrosis volume (g) in control patients by MRI (mechanism cohort); (C) AV Max (metres/second) determined by echocardiography (outcome cohort); (D) CT coronary calcium score (outcome cohort). ß values [95% confidence interval] represent change in the progression variable for each log unit change in cMyC concentration after adjustment in multivariate modelling (see Supplementary Tables S1-S3). *Adjusted for age, sex, glomerular filtration rate, AV_{max}, cardiac troponin, history of ischaemic heart disease, diabetes and hypertension; $^{+}$As above plus body surface area. ‡As above excluding AV_{max}.

Figure S5: Correlation between cMyC and myocardial cell death in 10 subjects from the mechanism cohort undergoing myocardial biopsy at the time of aortic valve replacement (sum of apoptosis, oncosis and autophagy). Correlation coefficient r=0.67, 95% CI 0.08-0.92, p=0.03.

Table S1: Linear regression modelling for the relationship between cMyC and left ventricular mass in patients with aortic stenosis (S1a) and healthy controls (S1b). Model 1 unadjusted; Model 2 adjusted for age and sex; Model 3 additionally adjusted for glomerular filtration rate and AV_{max}; Model 4 additionally adjusted for cardiac troponin; Model 5 additionally adjusted for comorbidity (ischaemic heart disease, diabetes mellitus and hypertension). ß values represent change in LV mass in univariate and multivariate analyses. ***p<0.001, **p<0.01, *p<0.05.

Table S2: Linear regression modelling for the relationship between cMyC and fibrosis volume in patients with aortic stenosis (S2a) and healthy controls (S2b). Model 1 unadjusted; Model 2 adjusted for age and sex; Model 3 additionally adjusted for glomerular filtration rate and AV_{max}; Model 4 additionally adjusted for body surface area; Model 5 additionally adjusted for cardiac troponin; Model 6 additionally adjusted for comorbidity (ischaemic heart disease, diabetes mellitus and hypertension). ß values represent change in fibrosis volume in univariate and multivariate analyses. ***p<0.001, **p<0.01, *p<0.05.

Table S3: Linear regression modelling for the relationship between cMyC and extracellular volume in patients with aortic stenosis (S3a) and healthy controls (S3b). Model 1 unadjusted; Model 2 adjusted for age and sex; Model 3 additionally adjusted for glomerular filtration rate and AV_{max}; Model 4 additionally adjusted for body surface area; Model 5 additionally adjusted for cardiac troponin; Model 6 additionally adjusted for comorbidity (ischaemic heart disease, diabetes mellitus and hypertension). ß values represent change in extracellular volume in univariate and multivariate analyses. ***p<0.001, **p<0.01, *p<0.05.

Table S4: Univariate correlations between log cMyC and baseline variables used for covariate adjustment in aortic stenosis patients (mechanism cohort).

Table S5: Cox proportional hazard modelling for the relationship between cMyC and all-cause mortality at a median follow-up of 11.3 years. Model 1 – including only aortic valve replacement as a time-varying covariate; Model 2 –additionally adjusted for age and sex; Model 3 - as Model 2 plus adjustment for maximum velocity across the aortic valve (AV

Max); Model 4 - as Model 2 plus adjustment for CT coronary calcium score. Values are hazard ratios (95% CI). ***p<0.001, **p<0.01, *p<0.05.

Figure S1

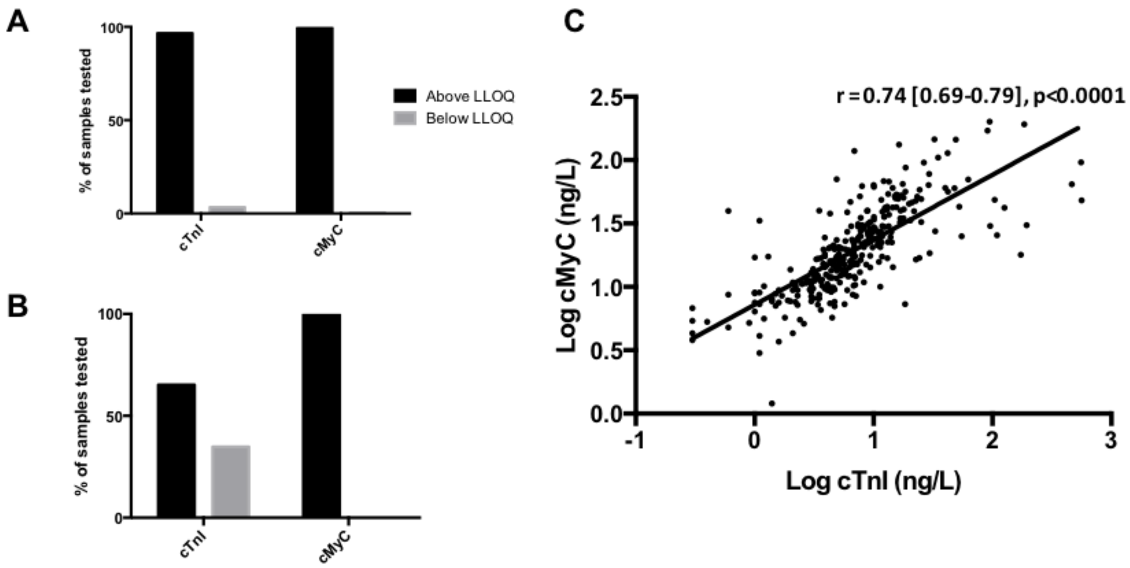
A
100
50
0
% of samples tested
cTnI
cMyC
Above LLOQ
Below LLOQ
B
100
50
0
% of samples tested
cTnI
cMyC
C
r = 0.74 [0.69-0.79], p<0.0001
2.5
2.0
1.5
1.0
0.5
0.0
Log cMyC (ng/L)
-1
0
1
2
3
Log cTnI (ng/L)

Figure S2

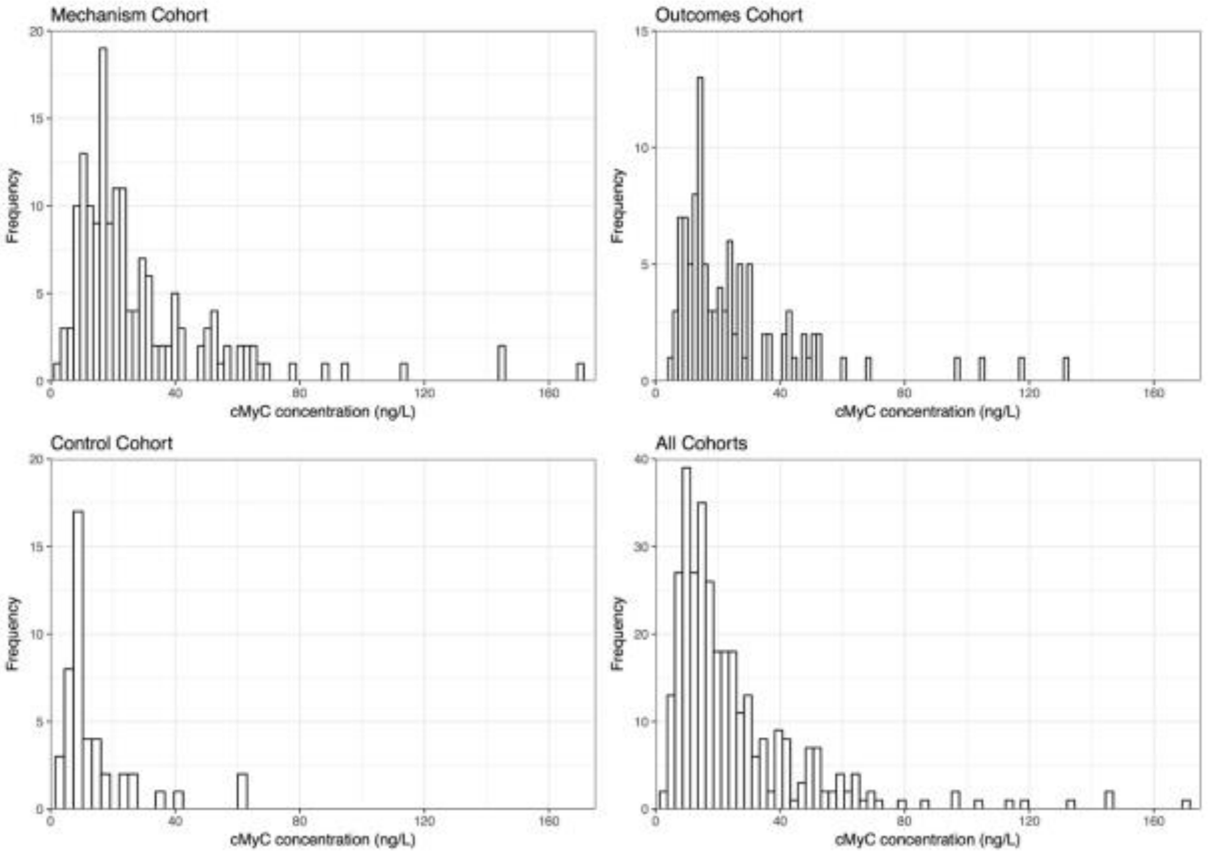

Mechanism Cohort
Outcomes Cohort
Control Cohort
All Cohorts
Frequency
cMyC concentration (ng/L)

Figure S3

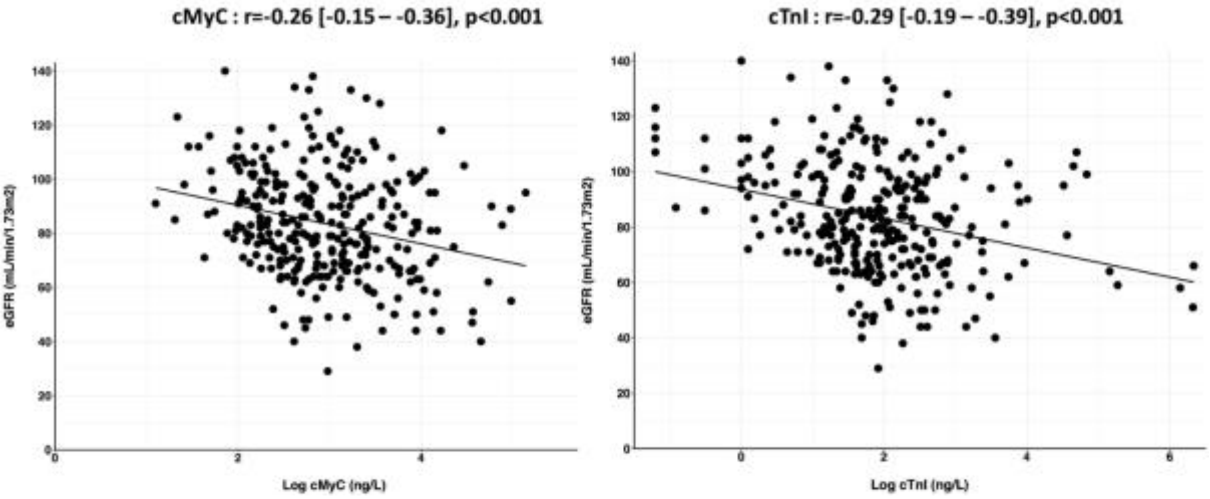
cMyC : r=-0.26 [-0.15 – -0.36], p<0.001
eGFR (mL/min/1.73m2)
Log cMyC (ng/L)
cTnI : r=-0.29 [-0.19 – -0.39], p<0.001
eGFR (mL/min/1.73m2)
Log cTnI (ng/L)

Figure S4

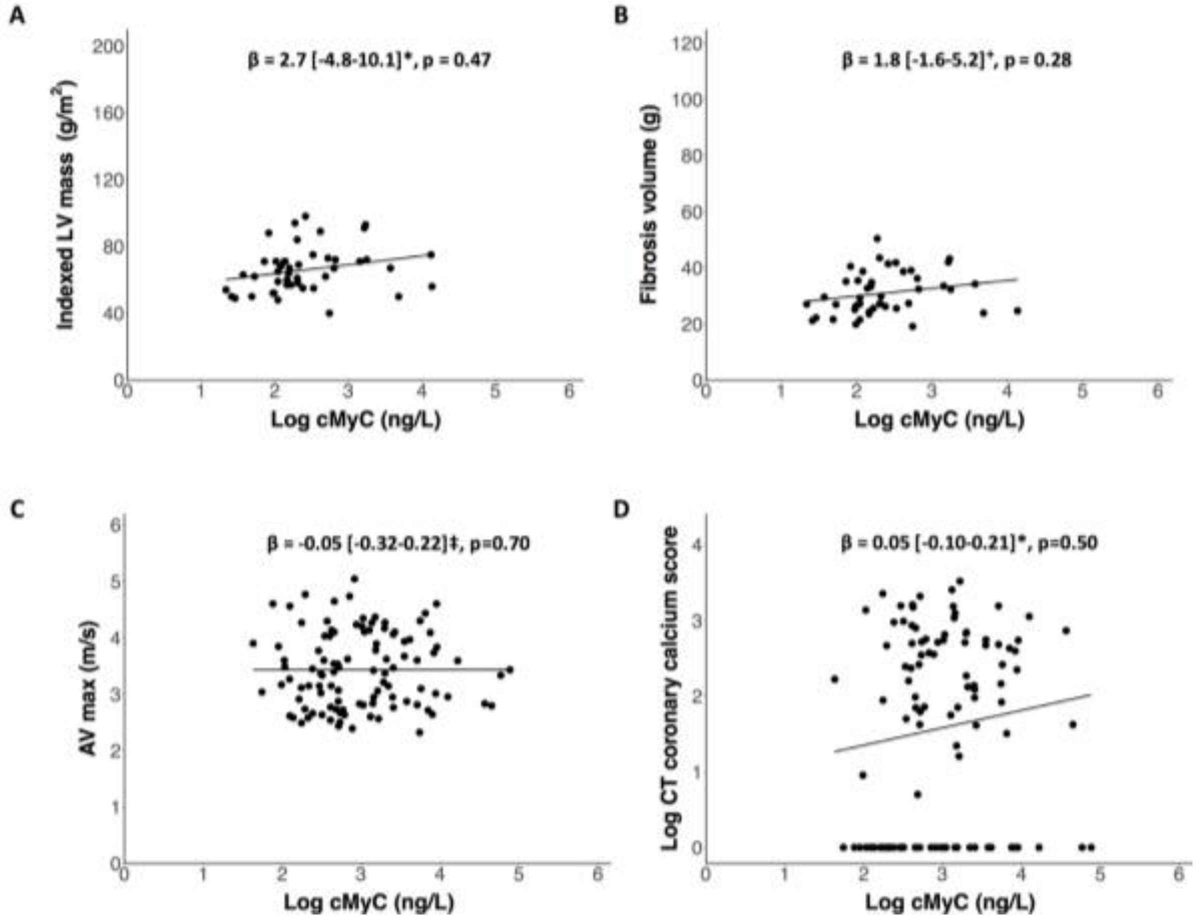
A
β = 2.7 [-4.8-10.1]*, p = 0.47
Indexed LV mass (g/m²)
Log cMyC (ng/L)
B
β = 1.8 [-1.6-5.2]*, p = 0.28
Fibrosis volume (g)
Log cMyC (ng/L)
C
β = -0.05 [-0.32-0.22]‡, p=0.70
AV max (m/s)
Log cMyC (ng/L)
D
β = 0.05 [-0.10-0.21]*, p=0.50
Log CT coronary calcium score
Log cMyC (ng/L)

Figure S5

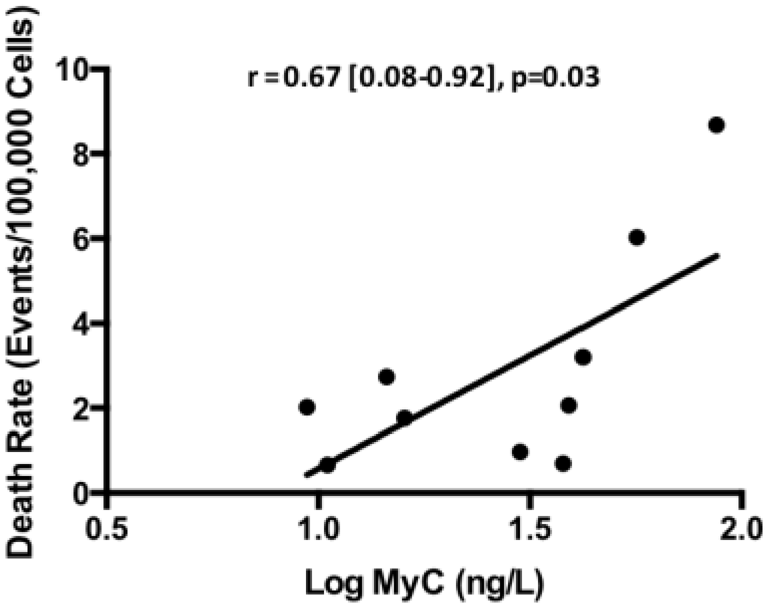
r = 0.67 [0.08-0.92], p=0.03
Death Rate (Events/100,000 Cells)
10
8
6
4
2
0
0.5
1.0
1.5
2.0
Log MyC (ng/L)

Table S1a: Linear regression modelling in aortic stenosis (LV Mass)

Variable	Model 1 ß	Model 2 ß	Model 3 ß	Model 4 ß	Model 5 ß
Log cMyC	16.8*** (13.0 – 20.7)	17.4*** (13.5 – 21.3)	14.0*** (9.7 – 18.3)	10.9*** (4.7 – 17.1)	11.0*** (4.7 – 17.3)
Age, per 10 years		-4.3*** (-1.9 – -6.7)	-3.3** (-0.8 – -5.7)	-3.3** (-0.9 – -5.7)	-3.0* (-0.3 – -5.6)
Male sex		10.4*** (4.6 – 16.2)	10.2*** (4.6 – 15.9)	10.6*** (5.0 – 16.2)	11.4*** (5.4 – 17.4)
eGFR, per fall of 10mL/min/1.73m^2			-1.4 (-2.9 – 0.1)	-1.3 (-2.8 – 0.1)	-1.3 (-2.8 – 0.2)
AV_{max}, per 1m^2			6.4*** (3.3 – 9.5)	6.6*** (3.5 – 9.7)	6.6*** (3.5 – 9.8)
Log cTnI				2.5 (-1.1 – 6.1)	2.5 (-1.2 – 6.2)
Ischaemic heart disease					-2.3 (-8.1 – 3.4)
Diabetes mellitus					-1.1 (-8.3 – 6.1)
Hypertension					0.0 (-6.2 – 6.2)
Adjusted r^2	0.32	0.42	0.48	0.49	0.48

*eGFR – estimated glomerular filtration rate. ***p<0.001, **p<0.01, *p<0.05.*

Table S1b: Linear regression modelling in controls (LV Mass)

Variable	Model 1 ß	Model 2 ß	Model 3 ß	Model 4 ß	Model 5 ß
Log cMyC	5.3<br>(-0.9 – 11.5)	4.2<br>(-1.9 – 10.2)	6.8*<br>(0.3 – 13.4)	3.1<br>(-4.4 – 10.6)	2.7<br>(-4.8 –10.1)
Age, per 10 years		-2.0*<br>(-0.2 – -3.9)	-0.5<br>(-2.6 – 1.7)	-1.1<br>(-3.3 – 1.1)	-1.8<br>(-4.1 – 0.6)
Male sex		12.3**<br>(4.5 – 20.1)	9.5*<br>(1.7 – 17.2)	9.5*<br>(2.0 – 17.0)	8.9*<br>(1.4 – 16.4)
eGFR, per fall of $10mL/min/1.73m^2$			-3.1**<br>(-0.8 – -5.4)	-3.0*<br>(-0.8 – -5.3)	-2.4*<br>(-0.1 – -4.8)
AV_{max}, per $1m^2$			1.9<br>(-12.6 – 16.4)	-0.1<br>(-14.3 – 14.2)	-7.8<br>(-24.7 – 9.1)
Log cTnI				3.9<br>(-0.2 – 8.1)	4.6*<br>(0.4 – 8.8)
Ischaemic heart disease					12.3<br>(-7.2 – 31.7)
Hypertension					4.0<br>(-5.1 – 13.1)
Adjusted r^2	0.04	0.25	0.33	0.37	0.39

*eGFR – estimated glomerular filtration rate. ***p<0.001, **p<0.01, *p<0.05. Diabetes excluded from modelling as there were no positive cases within the control group.*

Table S2a: Linear regression modelling in aortic stenosis (Fibrosis Volume)

Variable	Model 1 ß	Model 2 ß	Model 3 ß	Model 4 ß	Model 5 ß	Model 6 ß
Log cMyC	11.2*** (8.4 – 14.0)	11.7*** (8.9 – 14.6)	9.7*** (6.4 – 13.0)	9.7*** (6.6 – 12.7)	7.9*** (3.5 – 12.3)	8.0*** (3.5 – 12.6)
Age, per 10 years		-3.5*** (-1.7 – -5.2)	-3.0** (-1.1 – -4.8)	-1.7 (-3.5 – 0.1)	-1.7 (-3.6 – 0.1)	-1.4 (-3.4 – 0.7)
Male sex		7.2** (2.9 – 11.4)	7.5*** (3.2 – 11.8)	2.3 (-2.3 – 6.9)	2.6 (-2.0 – 7.2)	3.0 (-1.8 – 7.8)
eGFR, per fall of 10mL/min/1.73m^2			-0.5 (-1.6 – 0.6)	-0.8* (-1.9 – 0.2)	-0.8 (-1.8 – 0.3)	-0.7 (-1.8 – 0.3)
AV_{max}, per 1m^2			3.9** (1.6 – 6.3)	3.8*** (1.6 – 6.0)	3.9*** (1.7 – 6.1)	3.9*** (1.7 – 6.2)
Body surface area, per m^2				26.4*** (15.3 – 37.5)	26.0*** (14.9 – 37.1)	26.7*** (15.2 – 38.2)
Log cTnI					1.4 (-1.2 – 4.0)	1.4 (-1.2 – 4.0)
Ischaemic heart disease						-1.6 (-5.7 – 2.6)
Diabetes mellitus						0.6 (-4.8 – 6.0)
Hypertension						-1.0 (-4.8– 6.0)
Adjusted r^2	0.28	0.39	0.43	0.50	0.50	0.50

*eGFR – estimated glomerular filtration rate. ***p<0.001, **p<0.01, *p<0.05.*

Table S2b: Linear regression modelling in controls (Fibrosis Volume)

Variable	Model 1 ß	Model 2 ß	Model 3 ß	Model 4 ß	Model 5 ß	Model 6 ß
Log cMyC	2.8 (-1.0 – 6.5)	1.7 (-1.9 – 5.4)	4.1* (0.7 – 7.5)	3.8* (0.7 – 6.8)	2.1 (-1.4 – 5.6)	1.8 (-1.6 – 5.2)
Age, per 10 years		-0.9 (-1.9 – 0.1)	0.1 (-0.9 – 1.0)	0.4 (-0.6 – 1.4)	0.2 (-0.8 – 1.2)	-0.2 (-1.3 – 0.8)
Male sex		7.4** (3.1 – 11.7)	4.7* (0.7 – 8.7)	1.2 (-3.0 – 5.3)	1.0 (-3.1 – 5.0)	0.5 (-3.4 – 4.5)
eGFR, per fall of 10mL/min/1.73m^2			-2.3*** (-1.1 – -3.6)	-2.1*** (-1.0 – -3.2)	-2.0*** (-0.9 – -3.1)	-1.7** (-0.6 – -2.8)
AV_{max}, per 1m^2			4.4 (-3.0 – 11.8)	2.0 (-4.7 – 8.7)	0.9 (-5.7 – 7.6)	-3.6 (-11.2 – 4.0)
Body surface area, per m^2				18.5** (7.3 – 29.7)	20.0*** (9.0 – 31.0)	20.6*** (9.9 – 31.3)
Log cTnI					1.7 (-0.1 – 3.6)	2.1* (0.2 – 3.9)
Ischaemic heart disease						6.5 (-2.0 – 15.0)
Hypertension						2.6 (-1.4 – 6.6)
Adjusted r^2	0.03	0.25	0.44	0.56	0.59	0.62

*eGFR – estimated glomerular filtration rate. ***p<0.001, **p<0.01, *p<0.05. Diabetes excluded from modelling as there were no positive cases within the control group.*

Table S3a: Linear regression modelling in aortic stenosis (ECV%)

Variable	Model 1 ß	Model 2 ß	Model 3 ß	Model 4 ß	Model 5 ß	Model 6 ß
Log cMyC	1.2*** (0.7 – 1.7)	1.3*** (0.8 – 1.9)	1.5*** (0.8 – 2.1)	1.5*** (0.8 – 2.1)	1.3* (0.2 – 2.3)	1.3* (0.3 – 2.3)
Age, per 10 years		0.2 (-0.2 – 0.5)	0.2 (-0.2 – 0.6)	0.2 (-0.2 – 0.6)	0.2 (-0.2 – 0.6)	0.3 (-0.1 – 0.7)
Male sex		-1.4** (-0.6 – -2.3)	-1.4** (-0.5 – -2.3)	-1.3* (-0.3 – -2.4)	-1.3* (-0.3 – -2.4)	-1.3* (-0.2 – -2.4)
eGFR, per fall of 10mL/min/1.73m^2			-0.1 (-0.3 – 0.2)	-0.1 (-0.3 – 0.2)	-0.1 (-0.2 – 0.3)	-0.0 (-0.3 – 0.2)
AV_{max}, per 1m^2			0.0 (-0.5 – 0.5)	0.0 (-0.5 – 0.5)	0.0 (-0.5 – 0.5)	0.0 (-0.5 – 0.5)
Body surface area, per m^2				-0.3 (-2.8 – 2.2)	-0.4 (-2.9 – 2.1)	-0.4 (-3.0 – 2.2)
Log cTnI					0.2 (-0.4 – 0.7)	0.2 (-0.4 – 0.8)
Ischaemic heart disease						-0.2 (-1.2 – 0.7)
Diabetes mellitus						1.2* (0.0 – 2.4)
Hypertension						-0.7 (-1.7 – 0.3)
Adjusted r^2	0.10	0.16	0.16	0.15	0.15	0.17

*eGFR – estimated glomerular filtration rate. ***p<0.001, **p<0.01, *p<0.05.*

Table S3b: Linear regression modelling in controls (ECV%)

Variable	Model 1 ß	Model 2 ß	Model 3 ß	Model 4 ß	Model 5 ß	Model 6 ß
Log cMyC	-0.4<br>(-1.2 – 0.5)	-0.2<br>(-1.1 – 0.6)	0.1<br>(-0.8 – 1.0)	0.2<br>(-0.7 – 1.0)	0.5<br>(-0.5 – 1.5)	0.5<br>(-0.5 –1.5)
Age, per 10 years		0.2<br>(0.0 – 0.5)	0.4**<br>(0.1 – 0.7)	0.4**<br>(0.1 – 0.6)	0.4**<br>(0.1 – 0.7)	0.4*<br>(0.1 – 0.7)
Male sex		-1.5**<br>(-0.5 – -2.5)	-1.9***<br>(-0.9 – -2.9)	-1.3*<br>(-0.2 – -2.5)	-1.3*<br>(-0.2 – -2.4)	-1.4*<br>(-0.2 – -2.5)
eGFR, per fall of 10mL/min/1.73m^2			-0.4*<br>(-0.7 – 0.0)	-0.4*<br>(-0.1 – -0.7)	-0.4**<br>(-0.1 – -0.7)	-0.4*<br>(-0.1 – -0.7)
AV_{max}, per 1m^2			0.1<br>(-1.8 – 2.0)	0.5<br>(-1.4 – 2.3)	0.7<br>(-1.2 – 2.6)	0.2<br>(-2.1 – 2.5)
Body surface area, per m^2				-2.9<br>(-6.0 – 0.2)	-3.2*<br>(-6.3 – -0.2)	-3.1<br>(-6.3 – 0.1)
Log cTnI					-0.4<br>(-0.9 – 0.2)	-0.3<br>(-0.9 – 0.2)
Ischaemic heart disease						0.5<br>(-2.0 – 3.0)
Hypertension						0.4<br>(-0.8 – 1.6)
Adjusted r^2	0.00	0.21	0.27	0.32	0.34	0.31

*eGFR – estimated glomerular filtration rate. ***p<0.001, **p<0.01, *p<0.05. Diabetes excluded from modelling as there were no positive cases within the control group.*

Table S4: Univariate correlations between log cMyC and baseline variables (mechanism cohort)

Variable	β coefficient for log cMyC (95% CI)	p-value
Age, per 10 years	0.21 (0.12 – 0.30)	<0.001
Male sex	0.40 (0.16 – 0.64)	0.001
eGFR, per fall of $10mL/min/1.73m^2$	0.07 (0.02 – 0.13)	0.01
AV_{max}, per $1m^2$	0.36 (0.25 – 0.48)	<0.001
Body surface area, per m^2	0.18 (-0.43 – 0.79)	0.57
cTnI, per log unit increase	0.36 (0.32 – 0.41)	<0.001
Ischaemic heart disease	0.37 (0.14 – 0.59)	0.002
Diabetes mellitus	0.18 (-0.14 – 0.49)	0.27
Hypertension	0.29 (0.05 – 0.52)	0.02

Table S5: Cox Proportional Hazard Modelling (cMyC and all-cause mortality)

	Model 1	Model 2	Model 3	Model 4
cMyC, per doubling	1.49** (1.11 – 2.01)	1.26 (0.88 – 1.80)	1.26 (0.88 – 1.80)	1.27 (0.88 – 1.82)
AVR (time dependent variable)	0.68 (0.31 – 1.49)	0.75 (0.34 – 1.65)	0.78 (0.33 – 1.85)	0.75 (0.34 – 1.66)
Age, per 10 years		2.14 ** (1.69 – 2.71)	2.14 ** (1.69 – 2.71)	2.12** (1.65 – 2.72)
Sex, male		1.64 (0.70 – 3.81)	1.64 (0.71 – 3.82)	1.61 (0.67 – 3.89)
AV_{max}, per 1m/s increase			0.94 (0.53 – 1.67)	
CT Coronary Calcium Score, per 10 fold increase				1.02 (0.75 – 1.38)

Supplementary Material: Myocardial biopsy and histological analysis

Tissue Sampling

At the time of open heart surgery myocardial biopsies were obtained from the basal segment of the septum using a Tru-Cut needle biopsy gun. In order to minimise the chance of missed biopsies at least two samples per patient were collected. Immediately after collection, tissue was placed in buffered 10% formalin and subsequently embedded in paraffin.

Tissue processing

For apoptosis 7µm thick sections were deparaffinised in xylene, rehydrated through a graded series of alcohols and subsequently the DeadEndTM Fluorometric TUNEL System (Promega Co, US) was applied according to the manufacturer's guidelines. The system labels fragmented DNA which is a hallmark of apoptosis. After cell membrane permeabilisation the 3' OH ends of the cleaved DNA multimers were "tailed" with labelled fluorescin-12-dUTP by the Terminal Deoxynucleotidyl Tranferase enzyme. In addition all specimens were stained with 4,6-diamidino-2-phenylindole (DAPI) to adequately visualise nuclei which was necessary for proper image analysis.

For autophagy and oncosis formalin-fixed, paraffin-embedded 4µm thick tissue sections were cut. Slides were dried for 24 hours in a 45°C oven, deparaffinised in xylene and rehydrated through a graded series of alcohols. Slides were then loaded into a Celerus Riptide de-cloaking chamber (Celerus Diagnostics Carpinteria, CA , United States) where heat-induced epitope retrieval was performed using Novocastra Epitope Retrieval solution Ph6 (Leica Microsystems GmbH, Ernst-Leitz-Straße, Wetzlar, Germany). Slides were then loaded onto Leica Bond-Max automated immunostainer (Leica Microsystems GmbH, Ernst-Leitz-Straße, Wetzlar, Germany). For autophagy and oncosis ubiquitin and C9 mouse monoclonal antibodies at 1:3000 and 1:2000 dilution (Life Technologies, Carlsbad, CA, United States and

AbD Serotec, Kidlington, Oxford, UK respectively) were applied to sections at room temperature for 2 hours. The specificity of these antibodies was verified by omission. The presence of antigen was visualized using a 3,3'-diaminobenzidine (DAB) based Bond Polymer refine detection kit (Leica Microsystems GmbH, Ernst-Leitz-Straße, Wetzlar, Germany). Slides were counterstained using haematoxylin (in order to enable nuclei identification), removed from bond max, dehydrated, cleared and mounted with permanent mounting media (Pertex).

Histological image analysis

The TUNEL stained tissue samples were analysed using confocal microscopy with FITC and UV filter cubes. All measurements have been performed using 40x objectives. Apoptotic cells (co-positive for both DAPI and TUNEL) were counted manually on entire tissue sections. The total cell number present on each slide was derived from two sets of data: the total sample area and the number of cells positively stained with DAPI which was evaluated manually in three random areas of interest. Eventually the number of apoptotic cells was expressed as a percentage of the total cell number.

All C9 and ubiquitin stained slides images were acquired on the AxioScan Z1 (Carl Zeiss, Oberkochen, Germany) and analysed using Image-Pro Premiere 9.1 (MediaCybernetics, Rockville, MD, USA). In the first step the number of oncotic/autophagic cells was calculated using the counting toll after manual protocol adjustment. Cut off values of signal intensity, object size (area) and a roundness criterion was used to distinguish myocytes positively stained with DAB from artefacts. For Oncosis a pixel intensity of 0-163 on the Mono scale together with an object area range of 400-3000 square pixels and roundness criterion of 1-1.7 was applied. For autophagy the settings were as follows: a pixel intensity of 0-60 and an object area range of 200-7000 square pixels. In the second step the average cell area and the

total tissue area was measured using a threshold of 0-238 on the Mono pixel intensity scale. Finally the number of oncotic or autophagic cells was expressed as a percentage of the total cell count.

Cell counts

The median number of cells examined per patient was 115,161 [IQR 78,427 – 193,585] for oncosis, 109,355 [IQR 90,821 – 169,453] for autophagy and 216,320 [IQR 196,640 – 284,500] for apoptosis.

Example apoptotic cell

As demonstrated by confocal microscopy and immunofluorescence. A: DAPI; B: TUNEL; C: fused image demonstrating co-staining.

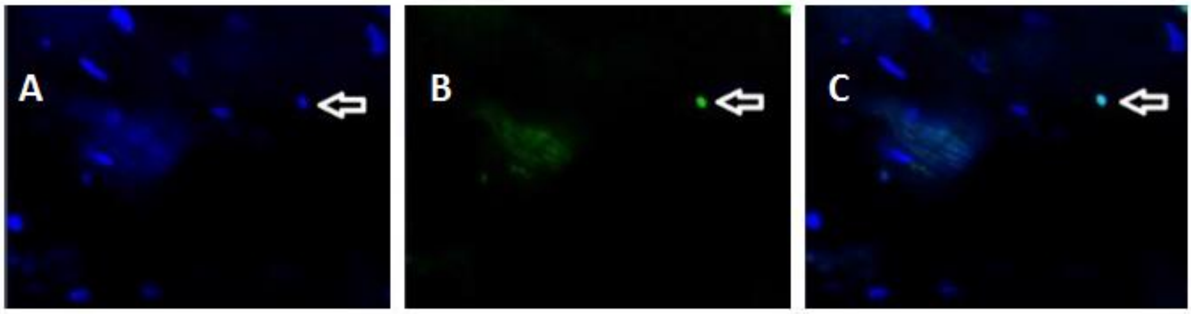

Supplement: Supplementary file 1 [file heartjnl-2017-312257supp001.pdf]
